# Structures and systems that promote nutrition security and climate adaptation in Puerto Rico: results from community-based system dynamics

**DOI:** 10.1017/S1368980025101080

**Published:** 2025-09-15

**Authors:** Uriyoán Colón-Ramos, Natalia Guerra Uccelli, Oscar Meléndez-Colón, Ana María García Blanco, César Ostolaza Santiago, Carla Rosas Pérez, Nicolás Gomez Andújar, Hector Tavárez, Lisa Poirier, Joel Gittelsohn, Michael W. Long

**Affiliations:** 1 George Washington University Milken Institute of Public Health, 950 New Hampshire Avenue Washington, DC 20052, USA; 2 Trito-Agro-Industrial Services, 521 Avenida Sagrado Corazón, San Juan 00915, Puerto Rico; 3 Instituto Nueva Escuela, 1101 Altos Ponce De León, Esq Paseo de Diego, San Juan 00925, Puerto Rico; 4 Asociación Pesquera de Culebra, Villa Pesquera Barrio Playa Sardinas I, Culebra 00775, Puerto Rico; 5 Mujeres de Islas, PO Box 358 Culebra 00775, Puerto Rico; 6 Universidad de Puerto Rico Mayagüez, 259 Av. Alfonso Valdés Cobián, Mayagüez 00680, Puerto Rico; 7 Bloomberg School of Public Health, Johns Hopkins University 615 N Wolfe St, Baltimore, MD 21205, USA

**Keywords:** Nutrition security, Group model building, Systems science, Climate adaptation, Food security

## Abstract

**Objective:**

This study aimed to develop a shared understanding about the drivers of nutrition security in Puerto Rico (PR) from the collective perspective of multi-sector stakeholders in the agri-food, environmental and the health/disease systems.

**Design:**

A participatory community-based system dynamics approach (group model building) engaged stakeholders during one 4-h workshop March 2023 (followed by two 2.5-h member checking sessions).

**Setting:**

San Juan, PR.

**Participants:**

Stakeholders (*n* 22) in PR representing the agri-food, environmental and health/disease systems from multiple sectors (commercial food retail and technology, food production, public servants, academia and civil society) participated in the workshop.

**Results:**

Stakeholders collectively framed nutrition security as an outcome of six interconnected subsystems exacerbated by climate change: (1) governance and public policy; (2) demographic change and rural disinvestment; (3) climate change and adaptive capacity; (4) local food production economy; (5) food culture; and (6) nutrition security and health. Stakeholders identified leverage points mostly focused on strengthening information flow within and across subsystems and expanding cross-sectoral collaboration (systems structures and elements). We identified three paradigms that have the potential to transform the system structure and function: ecological conscience, traditional and healthy food culture, and social cohesion.

**Conclusions:**

These findings deepened the collective understanding of systemic interdependencies that drive nutrition security as stakeholders identified locally feasible leverage points.

Nutrition security is defined as consistent and equitable access to healthy, safe and affordable foods that promote optimal health and well-being^(1)^, and it is widely recognised as an outcome of complex adaptive systems^(2)^. The behaviours of complex adaptive systems are determined by the system’s purpose (i.e. warding off hunger) and by their dynamic adaptation to influences within the system and exogenous to the system (such as climate change and food production)^(3)^. Despite the foundational focus on hunger and environmental degradation in the development of the field of system dynamics^(4)^, understanding of the system dynamics that lead to local nutrition security in the face of an exogenous, global challenge such as climate change is still in its early stages^(5)^.

Efforts to address nutrition security highlight the need to target multiple levels^(1)^ but do not sufficiently elucidate the system structure or function^(6)^. This is in part due to the fact that nutrition security is driven by the agri-food system, the environmental system and the health/disease system^(2)^ with multiple interdependent local and global factors from the genetic to national and international food markets, and global climate-driven extreme weather events^(6–8)^. Baker *et al.* argue that the political economy of food systems is undertheorised, calling for an integrated approach that incorporates both the distribution of power and resources over time, governance and policy strategies to address asymmetric power, and a system dynamics focus on interdependency, feedback and emergent properties^(9)^. Holman *et al.* posit that along with ongoing scale challenges, the focus of climate research and policy efforts on mitigation (i.e. reducing greenhouse gas emissions) and limited attention to adaptation (i.e. adjusting systems to reduce climate risk) have led to insufficient ‘adaptation models of isolated sectors’, or in other words, systems that fail to address the complexity required to adapt to feedback and interdependencies between systems or sectors^(10)^. Although using agent-based modelling or system dynamics modelling has been proposed as an approach to identify solutions to the ongoing adaptation problems^(11,12)^, community participatory research in systems modelling is uniquely suited to influence change at the local levels needed to address nutrition security^(13–16)^. Participatory modelling often results in identifying actionable plans and strategies and can help develop a sense of ownership and commitment to those plans^(17,18)^. Foster-Fishman and colleagues argue that understanding the different perspectives of the problem, examining the political, social and cultural aspects of a dynamic system, and emphasising the subjective nature of this process are critical first steps to transformative system change. The problem can then be examined in terms of the immediate and root causes maintaining the status quo; only then can transformative, multilayer leverage points can be identified^(19)^.

In light of the intractability of the joint problems of nutrition security and climate change, and the need for new analytic approaches in public health nutrition, the objective of this study was to develop a shared understanding of the underlying system structure among local stakeholders in Puerto Rico (PR), a territory of the USA that is highly sensitive and affected by climate change and nutrition insecurity.

## Methods

This study employed a community-based system dynamics (CBSD) approach to bring together stakeholders from multiple sectors and draw on their collective learning to understand the complex problem of food systems and climate health in PR. CBSD draws on community-based participatory research and system dynamics fields with the goal of strengthening participants’ systems thinking to elucidate the underlying drivers of community problems^(20)^. It is a particularly useful approach to acknowledge ‘traditional wisdom’ and the emic or insider perspective, considering health inequities in highly underserved communities. In this study, we used group model building (GMB), a participatory, iterative process within CBSD designed to elicit discussion through a series of scripted activities that build on each other. The output of this GMB process is a co-created visual map which qualitatively represents how stakeholders collectively think about causal relationships between key variables and the feedback loops that continue the status quo.

### Study setting and context

This work focuses on the archipelago of PR, deemed by some to be the oldest colony in the world^(21)^, where rapid urbanisation and a shift away from local, self-sustenance agriculture in the last 70 years has led to a sharp increase in food imports (> 85 % of total food consumed is imported)^(22)^. During Spanish, and later US rule, the agricultural lands were exploited for export monocultures of sugar, tobacco and coffee, but Puerto Ricans were prevented from accessing farms for growing foods^(23)^. Operation Bootstrap, supported by tax incentives from the USA for manufactured goods, spearheaded the rapid urbanisation and industrialisation that was seen in the mid-20th century^(24)^. In the most recent United States Department of Agriculture (USDA) agricultural census data available for PR in 2018, the number of farms decreased by 37·5 % in only six years since the last census in 2012. Total farmland also decreased by 16·6 %, while the size of the largest farms increased^(25)^. The decline in small farms in PR is consistent with changes seen throughout the USA but is occurring at a faster rate within an agri-food system unable to sustain its local population. Meanwhile, efforts to industrialise small-scale fisheries in PR also resulted in inconsistent fishers’ programmes and a decline in viability and sustainability of fisheries^(26)^. With the introduction of the US Food Stamp Program and its successor, the Nutrition Assistance Program, in 1974 there were significant increases in food expenditure and demand for foods. By 2020, the demand for foods was met mostly by imported products: local agriculture produced only 0·7 per cent of PR’s gross domestic product and supplied only 15 % of domestic food consumption compared with 59 % in 1951^(22)^. The population growth rates have been declining since the mid-1970s with sustained population loss since the economic crises in 2005 and exacerbated by extreme weather events which pushed working-age demographic groups to urban areas or to the US mainland^(27)^. As with other countries experiencing industrialisation and urbanisation in the context of a global food system, PR has been experiencing a nutritional transition with dietary patterns characterised by a mix of traditional root crops as well as ultra-processed foods high in refined sugars, saturated fats, and animal protein, and related cardiometabolic risk factors^(28)^. Fast food venues are widely accessible^(29)^, and consumption of foods away from home has been associated with lower dietary quality in PR^(30)^. Today, diet-related chronic diseases are highly prevalent and people die from diabetes at twice the rate than Puerto Ricans living in the USA^(31)^.

In addition to degrading the trust of Puerto Ricans in their municipal, local and federal governments^(32)^, exposure to climate change also degraded the soil’s capacity to absorb rainfall and provide bioavailable nutrients to crops, which have led to increased irrigation and use of fertilisers, herbicides and pesticides, causing further depletion of groundwater supplies and waste, soil and water management crises^(33,34)^. In the last decade, citizens in PR have experienced hyperactivity of natural disasters which have coincided with long-term deteriorating infrastructure, a reduction in essential public services and a shrinking job market due to the political and economic incentives, as well as neglect, that drive the governing structure of US territories^(35,36)^. In a 2023 international poll, Puerto Ricans were among the most worried about climate change^(37)^, likely because they have collectively experienced long-drawn recoveries from extreme weather events, such as Hurricane Maria in 2017 which devastated the infrastructure, caused serious interruptions in the food supply chain and resulted in nearly 3000 deaths^(23,38)^.

These crises are often dealt with in unconnected silos. For example, concerns about food insecurity sparked policy investments to increase local food production to 50 %^(39)^, but if these initiatives fail to address the underlying drivers of nutrition insecurity, they will result in deepening climate crises and inequities in nutrition and health.

### Researcher positionality

The crises detailed above have strengthened grassroots movements to address the co-vulnerabilities of poverty, agroecology, nutrition and health in PR^(34)^. The work presented in this manuscript is the result of a collaboration between researchers in PR and the US mainland, leaning on decades of grassroot efforts that rely on organised citizens, non-governmental organizations and local producers to adjust and adapt, sparking innovation along the way. The academic partners are from public health research institutions and had previous experience with community-engaged research and participatory approaches, with the development of community–participatory interventions in public health nutrition and in CBSD approaches to build capacity in systems thinking among stakeholders. The community partners include PR’s only virtual farmers market enterprise with a network with > 400 local food producers; a commercial organic waste and management company with a network of > 500 individuals and 25 commercial users with ties to the PR Food Bank; and a non-governmental organization focused on participatory action research in the public school system in PR with > 10k teachers, families with children in their network. The core modelling team (CMT) that designed and led this project was composed of these community and academic partners (all coauthors of this study). The CMT sought to privilege Puerto Rican voices and worldviews through the CBSD approach. Over the course of this study, the CMT worked closely together to design, implement and analyse the qualitative data from this CBSD.

### Sampling strategy and participants

The CMT used a purposive sampling strategy to yield diverse perspectives from actors who were local champions and changemakers (i.e. individuals who will likely need to be involved with sustainable changes and who were already involved in ongoing changes) in the agri-food, environmental and health/disease systems. Across these systems, the CMT sought to represent diverse sectors, including commercial food retail and technology, public servants in government and nongovernmental organizations, food production, research entities and civil society. To identify and recruit participants, the CMT relied on their professional networks and used a snowball technique, asking who else would we seek or talk to about this topic, inviting potential participants sequentially (via email and phone calls) until at least two individuals from each sector confirmed participation. A total of twenty-two participants joined a 4-h workshop held in-person in the metropolitan area of San Juan, PR (Table 1).

**Table 1.** Number of stakeholders (in parenthesis) by sector who participated in the GMB workshop to develop a shared understanding of system dynamics driving nutrition security in the face of climate change in Puerto Rico. The workshop was held in March 2023 in San Juan, Puerto Rico (PR) (*n* 22 participants)

Sectors				
Commercial food retail and technology	Public servants focused on health and nutrition (non-governmental and government)	Local food production	Research and academia	Civil society
Food cold chain and distribution (1)	Medical society (1)	Farmers (2)	Environmental scientist (1);	Consumer: participant in US federal assistance programme WIC – special supplemental nutrition programme for women, infants and children) (1)
Supermarket representative (1)	Food and nutrition non-profit organisations (3)	Dairy producer (1)	Environmental economist (1)	Consumer: participant in federal and territory Nutritional Assistance Program (NAP) (2)
Software technology (2)	USDA food and nutrition (2)	Agronomist (1)	Economist (1)	Consumer and Medicare recipient (1)
		Aqua culturist (1)		

GMB, group model building.

### Data collection

The GMB workshop was held in-person in San Juan, PR, in March 2023. The workshop lasted 4 h; light refreshments were provided, and participants were compensated with a $120 gift card for attending. The CMT grouped confirmed participants into three small groups (6–7 participants in each group) with representation of diverse sectors in each group. The CMT guided the small groups through a series of established and publicly available scripted activities developed by members of the CBSD field (see Table 2 adapted from Scriptapedia^(40)^). These activities are designed to elicit both divergent and convergent perspectives from the groups and produce outputs that build on each other to ultimately create a visual representation of the variables that the group considers most important, and how they relate to each other (a causal loop diagram, or CLD). Participants worked in their small groups to develop their own CLD **(**refer to see online supplementary material, Supplemental Appendix A) to depict their shared understanding of the system dynamics driving nutrition security and climate change before each small group shared their CLD with the entire group and discussed similarities and differences.

**Table 2.** Scripts, functions and outputs that build on each other, employed in the GMB workshop to develop a shared understanding of system dynamics driving nutrition security in the face of climate change in Puerto Rico. Scripts (adapted from Hovmand *et al.* (2011))^(40)^

Scripts	Functions	Outputs
1. Hopes and fears	Establish a set of values that embrace diverse perspectives, transparency across all participants.	List of participants’ hopes and fears regarding discussion process
2. Food and nutrition insecurity in Puerto Rico over time	Initiate mapping by generating multiple factors as potential drivers of the problem of food and nutrition insecurity in Puerto Rico. Begin to generate narratives to explain current community conditions, based on historical trends and environmental, climate change factors	Candidate factors for CLD
3. Convergent dots	Sort through many possible choices and select those that are most important to group	Prioritised factors
4. Connecting circles	Introduce concepts of causal connections and feedback relationships in a system for increased and sustainable food and nutrition security in Puerto Rico. Make linkages between variables across sectors explicit.	Connecting circles per group
5. Causal loop diagram	Synthesise multiple perspectives of the problem and reveal new insight into improved food and nutrition security in sustainable ways, keeping grounded in the experiences of users.	One CLD per group
6. Action ideas (leverage points)	Identify and qualify action ideas according to perceived feasibility (what is already happening) and opportunities (what can be done).	Prioritised list of leverage points

GMB, group model building; CLD, causal loop diagram.

#### Leverage points

Completed CLD from each small group were taped to the wall, and participants were asked to use their small CLD to identify solutions (*Can we identify where to intervene to counter the threats to food and nutrition security in PR?).* To anchor the discussions, participants were asked to think about short- and long-term actions: things that were already happening on the ground (short-term actions) as well as solutions that seemed unfeasible and challenging at the moment. As they mentioned each solution, and discussed it out loud with the larger group, they were asked to describe the solution as hard to do or easy to do (and to describe if anything was already happening regarding that solution in PR), and whether it would reach a lot of people or fewer people. This was documented in notes.

All group discussions were conducted in Spanish, audio-recorded and transcribed verbatim. All artefacts resulting from the scripted activities were photographed, collected and organised according to activity.

### Data analysis

Within 24 h after the GMB workshop, and in line with best practices for qualitative research^(41)^, the CMT met to debrief and consolidate their field notes and synthesise the small group CLD into one using Stella Architect, a visual programming language for system dynamics modelling. During this initial analysis, the CMT (1) first merged overlapping factors and connections from the three small-group CLDs; (2) noted any factors, connections and subsystems where there was no overlap and included those as well in the synthesised CLD; (3) reviewed the remaining artefacts (i.e. products) that resulted from the GMB scripted activities to note any details (feedback loops and variables) that may be missing from the visual depictions of small-group CLD and noted those in a separate document. The artefacts were filed by categories of activities, and once the audio files from the group discussions were transcribed verbatim, two Spanish-speaking research assistants trained in qualitative data analysis reviewed the transcripts to extract exemplary quotes for each one of the feedback loops and connections that had been identified in the synthesised CLD. Subsequent reviews of the transcripts occurred in order to identify variables and their relationships that were not explicitly drawn in the CLD but that were discussed in the small groups during the workshop. The CMT met weekly for 4 months to review the quotes and the synthesised CLD and did another pass at the transcripts, field notes and artefacts to ensure that they accurately captured what was conveyed in the synthesised CLD. Revisions were made to the CLD continuously with a focus on comprehensive and integrated representation of participants’ understanding of the pre-existing systems, primarily adding implied or missing polarities or connections. By mapping participant quotes to the factors, connections and feedback loops, the CMT added richness to the qualitative CLD and rigour to the process of developing the consolidated CLD.

Following the creation of the merged CLD, the CMT members reassessed the factors and connections in the model with the goal of highlighting the feedback loops that participants had drawn and that could be gleaned from the transcripts. In this iterative process, feedback loops were identified as seeds of subsystems of the overall system structure using our CMT broad experience working within the local system (i.e. food retailer, commercial scale composting, local education and evaluation).

#### Leverage points

All action ideas elicited during the GMB were listed, along with a description of what is currently happening in the territory, and where the opportunities for action were. The CMT further categorised each idea by referring to Meadows’ 12 points of effective places to intervene within systems^(42)^ (later summarised into five by in the Intervention Level Framework – ILF)^(43)^.

#### Techniques to enhance trustworthiness

Once the merged CLD was completed and checked by the CMT against artefacts, transcripts and notes, the CMT invited workshop participants to attend a virtual member checking. The action ideas (leverage points) that were elicited during the workshop were presented back to the participants via email in a newsletter the day after the workshop and discussed during the virtual member checking session. A total of thirteen participants attended two sessions of member checking, each lasting 2·5 h, and provided feedback on the synthesised CLD. The feedback received was that the subsystems needed to be simplified and that a hybrid CLD (with stocks and flows) was more easily understood. We then focused our analysis of the system structure on identifying stocks and flows, better connecting subsystems representing the potential delay in changes in systems when stocks need to be replenished. Stocks and flows are a type of diagram that depicts quantities of a stock accumulating over time, and a valve attached to the flow pipes that represent the rate at which something enters or leaves the stocks. Following these member checking meetings, the CMT further revised the CLD into its current form presented here.

## Results

**Figure 1.**
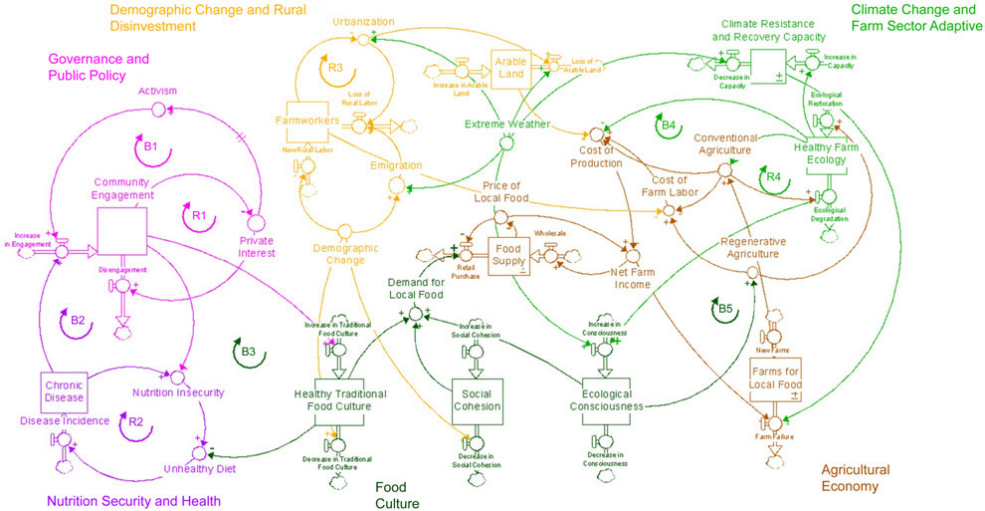
Hybrid causal loop diagram with six subsystems representing the underlying drivers of nutrition insecurity in the context of climate change in Puerto Rico. This visualisation resulted from a group model building workshop that was held in Puerto Rico, March 2023, in which 22 stakeholders from diverse sectors participated.

Figure 1 shows the final hybrid CLD (with stocks and flows) that depicts how multisectoral participants visualised the factors that drive nutrition security in PR and feedback loops between six subsystems: governance and public policy; nutrition security and health; demographic change and rural disinvestment; climate change and adaptive capacity; local food production; and food culture. Below, we include a description of the reinforcing and balancing feedback loops. Reinforcing loops are those for which a change (either positive or negative) in the level of a variable propagates through the loop and ultimately reinforces the initial change and are labelled R1–R4. Balancing loops, in which a change in the initial variable leads to an opposite change at the end of the process, are labelled B1–B5. Quotes from the discussions are included to exemplify loops; these quotes have been translated to English and edited for clarity and conciseness.

### Subsystem 1: Territory governance and public policy

Participants shared their understanding of the colonisation processes that created the inequities present today, and the inability of the territory to self-govern permeated the discussions around food availability and nutrition security. Within that context, the discussion around the decrease in local food production (which was the original reference problem provided) was centred on the territory and represented as a subsystem with two loops.

#### The ‘Private Interests’ reinforcing loop (R1)

According to participants, the underlying driver of expensive inputs for food production and the repercussions in nutrition was public policy driven by the private economic interests of a few influential and large companies.

*‘[…]based on external public policies that have repercussions on the local agricultural production and obviously also the nutrition or what can be your local products[…] Puerto Rico has two agricultural taxes that [were meant to protect private interests of sugar and coffee plantations and today] make no sense…’*

Private interests in governance and policy were also exemplified by the costs of agricultural inputs. Participants discussed the perceived paradox of how in PR, one of the largest producers of seeds in the world, it is still challenging to purchase seeds for local farming – a problem that became more evident in the aftermath of Hurricane Maria.

*‘Even if you have 200 cuerdas [a ‘cuerda’ ∼ to 0·971 acres of land] to farm celery root, where do you get the seed from? […]There is a public policy [referring to Act 62 of 2009 that allows multimillion dollar companies to develop agricultural biotechnology enterprises] that benefits multinationals to develop seeds but it does not benefit the local farmers’.*

The reinforcing loop of private economic interests becomes stronger without a balancing loop between citizen engagement and activism, as described below.

#### Activism balancing loop (B1)

Increases in the influence of private economic interests of a few influential companies to the detriment of Puerto Rican communities eventually caused social and political unrest and was thought to lead to citizen engagement.

*‘It has to do with citizen engagement. Everyone is doing something but unfortunately the way that the [.] leaders respond is through public pressure; a lot of public pressure. So, I think that we would have to start to see how we can make these conversations public and how to create public urgency so that the government and other decision makers can see the implications [their decisions] have’*.

### Subsystem 2: Demographic change and rural disinvestment

#### Rural labour spiral reinforcing loop (R3)

Participants discussed the expansion of urban developments and urban sprawl that spilled to the edge of city boundaries, even to areas that were supposedly protected by natural reserves. One GMB participant stated: *‘There are areas where agriculture can be done, but they are overdeveloped. You see that the city is falling apart but they [developers] still want to develop outside the city’*. Urbanisation led to a decline in local food production and a failing food economy: *‘Farmers are condemned to poverty. Without subsidies, the farmer cannot survive or earn a living wage for their family. It is not a viable career unless they diversify harvest and production’*. Rising temperatures coupled with underpaid, strenuous outdoor farm labour were cited as reasons why farming was devalued in society. As a result, farm labour supply is low. Participants discussed how this contributed to marginalisation and social and income inequality, potentially leading to higher crime and affecting people’s social well-being. Although a balancing loop was not identified, some participants suggested that guest-worker programmes would be the most effective near-term solution to rural labour shortages.

### Subsystem 3: Climate change and adaptive capacity

#### Farm ecology reinforcing loop (R4)

Limited workforce and farm labour were also inversely associated with adopting regenerative and climate-adaptive agriculture. As this was also the experience for coastal fisheries, the term ‘food production unit’ will be used to be more inclusive. A GMB participant explained the need for conventional agricultural practices to make work more manageable: *‘If herbicide is not used, someone has to be there with a machete working to get rid of weeds. If there were 500 acres of land, that would be a lot of work and constant labor’*. This cycle (R4) portrays how a limited food production unit reinforces the use of conventional practices (e.g. in the case of farming: deforestation, tilling, monocropping, chemical fertilisers, pesticides and groundwater irrigation), which further degrades the ecology and, in turn, requires even higher investments in conventional practices that further degrade the environment.

### Subsystem 4: Local food production

#### Farmers’ dilemma balancing loop (B4)

Farmers facing low margins and high risk pursue conventional practices to control costs. Over time, these practices can sufficiently reduce soil and ecological health, require more inputs that erode net income and increase the risk of farm failure. Therefore, the deteriorating farm ecology cycle is only balanced by the increasing cost of local production, which ultimately leads to high rates of local farm failure. This is, thus far, what has been happening in PR with high rates of farm failure and declining local production:

*‘This is a terrible cycle: local production goes down, price increases, consumption goes down, so less food is produced. This cycle has led us to [a decrease to] 15 % of local food production’*.

### Subsystems 5 and 6: Food culture and nutrition security and health

Some participants explained that, historically, local food production was insufficient to feed the growing population, eventually changing people’s eating preferences, to the point where younger generations were unfamiliar with local food sources and disconnected from local food production in a reinforcing feedback loop. As stated by participants: *‘… when they begin bringing more things from outside to be able to feed everyone, the Puerto Rican palate changes. […] The lack of knowledge of local produce creates a demand for imported products’.*

Further, the daily struggle of working multiple jobs and endless hours just to make ends meet in the modern-day context (or ‘*La Brega*’ as participants called it) led to a fast lifestyle, stress, quick food fixes and degradation of health: *‘Something that impacts local food consumption is the fast lifestyle that we have. The two parents are working, get out late and pick up their children. They have to give [them] something quick [to eat]’*. The increasing cost of living makes it even harder to disrupt this cycle. The CMT summarised this as the ‘Health-Poverty Trap’ reinforcing loop (R2), best expressed by this quote: *‘[…] Nutrition insecurity is not due to dietary choices. It is an issue of poverty and lack of resources’*.

#### Chronic disease crisis balancing loop (B2)

Similar to the activism loop (B1), this balancing loop describes how citizens were facing intolerable conditions and crises are the underlying drivers of citizen engagement and activism. Citizens will eventually demand and develop structures and systems to support their health. Some of this is already actively happening via collective action of citizens, tackling individual variables or loops within the system, but what was needed were synchronised efforts to address nutrition, local food production and environmental health simultaneously in the context of climate change.

*‘We must have a shared vision; [to have] everyone speak the same language, be seated at the same table, and in one common direction; because well, each one of us is in a state of survival in Puerto Rico. Each trying to live and do what they can but, working together, and joining all who are willing, a lot more can be done’*.

#### Leverage points

Table 3 presents the ideas that were proposed as intervention and action items based on what was feasible (depending on what was already happening in PR to their knowledge) and on the level of where to intervene in the system (Table 3). Most of the leverage ideas addressed structural elements of a subsystem (i.e. providing financial incentives for local/imported food production) and items related to the system structure and flow of information (i.e. addressing who does and does not have access to what kinds of information). Examples include using schooling and education to improve information flow across sectors and subsystems and calling for increased connections and collaboration across sectors and disciplines, thereby expanding boundaries across subsystems. While these would be considered mid-level in terms of effectiveness^(44)^, there was also mention of paradigm shift (or deep system values, often implicit) across the subsystems: the value of local farmers and local food production, the value of local diets and the value of ecological interventions. During the member checking, these leverage points were used to structure the CLD into three underlying paradigms that need to be stocked or replenished in order to feed back into demand for local foods that were ecologically conscious and healthy. For example, participants agreed that ecological consciousness was on the rise among people in PR. Ecological conscience was expected to increase consumer demand for products that had been grown using climate-smart practices. If those products are local, they are expected to offer a balance to the reinforcing loops of farm ecology and farms for local foods. However, waiting for ecological conscience to increase consumer demand may be too little too late for nutrition insecurity and climate health. One way to reinforce social cohesion and its translation into policy is through a ‘Healthy Traditional Food Culture’, which can emerge from the loop of a chronic disease crisis and the high costs of diet-related chronic disease morbidity and mortality. Social cohesion, and policies and programmes that support the purchase of healthy traditional foods and the promotion of a healthy traditional food culture, is expected to lead to reductions in unhealthy diets and lifestyle in a balancing loop to the subsystem of Nutrition Security and Health (Healthy Traditional Food Culture B3), as well as increasing demand for local food products. The final model shows that together, if and when these three values are strengthened, they are expected to lead to increased demand for local foods that are nutritious/healthy and better for the environment, eventually disrupting the cycle of degradation of population and planetary health.

**Table 3.** Leverage points identified by participating stakeholders during a GMB workshop to develop a shared understanding of system dynamics driving nutrition security in the face of climate change in Puerto Rico, held in San Juan, PR, on March 2023. The core modelling team organised each leverage point by effectiveness level according to ‘where to intervene on the systems’ (Malhi *et al.* 2009; Meadows 1999)^(43,44)^

Effectiveness level (ranked from least to most effective according to Meadows, 1999)	Leverage points	What is currently happening?	Where are the opportunities for action?	Exemplary discussion quote(s)
Level 6: Information flow across subsystems	Educational curricula for schoolchildren. Feasible Large Reach	Various efforts, funded by private foundations and/or federal government programmes (i.e. farm to school, or nutrition or climate) but not the nexus of climate, food and nutrition.	Synergise and coordinate efforts across sectors. Long-term goal: new generation that appreciates producing, consuming local foods that are healthy for themselves and the planet.	*‘Youth don’t know what yautía is. They don’t know what a malanga is. White yautía… they cannot buy the products we’re growing if they don’t know what they are’*
Level 6: Information flow within and across subsystems	Marketing and promotion to consumers. Feasible Potential large reach	Various – in an uncoordinated fashion touting eco-friendly or local.	Coordination, data about the convergence of regenerative agriculture and local products regarding each product, use of social media. In the long run, this is intended to increase demand.	*‘Lack of knowledge about the products and local brands. […]There is a short-term solution: a promotional campaign’*.
Level 6: Information flow across subsystems	Narratives to inspire an influence on public policy. Somewhat feasible. Reach: Unclear	Already happening in some areas.	Bolster and create content of the narratives across sectors Create a strategic policy plan proposal.	*‘One part of this [GMB] initiative that is important, is thinking about how to create a narrative that reaches that person who is creating public policy. And at the same time create more democratic content that inspires people to demand that public policy… It’s an interdisciplinary strategy… We are not only thinking about content but also in action’*
Level 6: System structure (collaboration)	Amplify and coordinate strategically the efforts and results in this topic of nutrition, food production and climate. Easy to do. Medium reach.	Currently limited to personal connections	Development of a food council to exchange data and information	*‘The disposition of many sectors to work exists… there are many initiatives… There has to be more cohesion between initiatives, for example, here they are represented’*
Level 5: Structural elements and system rules within a system	Invest in and protect local farming. Hard to do. Reach: large.	Extremely limited investment in local agriculture.	Taxes to protect against imports, especially in line with seasonal local products. Advocacy for local production (perhaps through a food council)	*‘If you invest in the farmer… imported food is cheaper than local food[…] participation in NAP [Nutrition Assistance Program], the farmer’s income counts, and they can only be on the payroll for 6 months. So NAP is a disincentive to work in agriculture as well […]And the other is the economic situation. If you pay me $9 an hour as a farmer, or $8·50. They don’t qualify for health insurance… or they tell them ‘payment in cash’, and the farmer cannot demand their worker’s rights… it’s like a cycle’*
Level 5: Structural elements and system rules within a system	Increase labour force for local food production. Hard to do. Medium reach.	Currently, there are financial incentives to import labour for agriculture.	Eventually, prepare for the demand in local products by raising against the negative stigma of working in local agriculture and farming.	*‘Instead of importing seeds, I’d import labor. […] you need labor. You have the seeds, but you don’t have who to help to move the oxen’*
Level 1: Paradigm	Hard to do.	Emerging but limited.	Value the local farmer.	*‘Well, I think that the negative perception of being a farmer is about social status. A lot of people say that working in agriculture is like moving backward; like going back to 1940’* *Emerging: [Discussing the new trends in young people farming] ‘Farming is not a hobby. And the farmer must comply with a series of practices: production, preparation of soil, marketing, safety of products, and the preparation of products. Here, things are done because farms are inherited and because [that is how] it was done that way and [now] that is the practice, but there is no culture’*
Level 1: Paradigm	Hard to do.	Emerging but limited.	Value traditional local foods for the diet.	*‘There is a social component with the imported diet, not local’*
Level 1: Paradigm	Hard to do.	Emerging but limited.	Value and invest in ecology.	*‘I think that today, there are a lot of people who are environmentally conscious. More than one thinks…’*

GMB, group model building.

## Discussion

Our findings contribute to the limited literature that uses participatory systems science approaches to *integrate* multiple perspectives to develop a shared vision about the drivers of *local nutrition security* in the *global context of climate change* ^(43,45)^. System dynamics methods are widely recognised as powerful tools for engaging community stakeholders into collective action^(17)^. The process of group modelling of systems can lead to changes in mental models of group participants, which increase the likelihood that stakeholders commit to implementing changes after participating in the GMB, depending on their ability to participate in the modelling, process information from different organisations and stake in the problem discussed^(17)^. According to the framework for transformative systems change proposed by Foster-Fishman *et al.*, a crucial first step towards transformative systems change is developing this shared understanding of the system structure and system function among multiple actors^(19)^. Therefore, a strength of this study is the inclusion of local perspectives from actors that are actively involved in aspects that are relevant to nutrition security through public service, grassroot movements or commercial initiatives. The inclusion of these insiders’ perspectives and iterative checks of the model allowed the participants to identify leverage points at multiple levels, with most ideas concentrating around structural elements and system structure (i.e. integrated curricula, information flow within and across subsystems, financial incentives and protections). Proximal structural elements and system structure tend to be the most popular ‘places to intervene’ in a system because these tend to be discrete efforts, planned for a specific period of time and easy to design, monitor and evaluate within a period of time^(43)^. In fact, public health nutrition efforts have a long history of addressing structural elements and system structure (i.e. information flow, design monitoring and evaluation of a curriculum or a distinct policy), partnering with local food production, such as local farmers to promote the intake of fruits and vegetables, especially among low-income communities^(46)^. These efforts tend to be episodic (i.e. planned and dependent on external funds for a disclosed period of time), rather than organic, internally motivated, continuous and sustained^(19)^. The latter are more challenging to achieve because they seek second-order changes that call for paradigm shifts (i.e. transformative changes in how things are perceived and done within a specific context)^(17)^. Indeed, according to Johnston *et al.*’s review of twelve obesity studies in North America, studying and addressing the underlying beliefs, values and experiences in a system is a vastly understudied area: with only 2 % of recommendations addressed the paradigms driving system function^(47)^.

Shifting paradigms or values related to social cohesion, ecological consciousness and traditional healthy diets is rarely done by episodic and short-term policies and intervention, but rather by a shared history and culture that creates a shift in mental models^(19)^. The leverage points identified in this study point towards needed shifts in the way people think about, and address, the problem of nutrition security in PR. Thinking and doing are highly linked, and in the context of the territory, the people who think about the problem are empowered to do something about the problem. The people who were invited and participated in the GMB both are thinking about the problem and are also more likely to do something about the problem: to implement a change within their organisations or departments; and to continue to think about problems in ways they had not thought about before, perhaps involving other sectors.

The findings of this study emphasise the need to address the underlying paradigms that currently devalue local food production, healthy traditional diet and ecological practices^(19,48)^. The proximal elements of the system structure that were identified could not achieve transformative change without addressing these underlying paradigms, related to the purpose and goal of the status quo of the system as is. One could argue the system was originally designed to maximise profit and ward off hunger by maximising its provision of calories^(22)^. In the editorial of the special issue of political economy of healthy and sustainable food systems, Baker *et al.* emphasise studies that show how trade liberalisation resulted in the expansion of, and investment in, transnational food corporations that produce and market ultra-processed foods and shape beliefs about what foods are socially desirable^(9)^. Determined by the purpose for which they were once designed, complex adaptive systems produce their own pattern of behaviour over time, adapting in response to exogenous global factors and external and internal influences^(42)^. Some argue that the expansion of global food markets devalue the livelihoods in local food systems and healthy, culturally appropriate foods^(9)^. Nonetheless, our findings suggest that experiencing extreme weather events and a rising prevalence of diet-related chronic diseases in the context of a health and financial crisis have re-awakened an interest for traditional cuisine, the environment and health in PR.

The question of who has decision-making power here is an important one. Baker *et al.* have also argued that power to transform food systems has shifted away from the national (to inter-governmental organisations at the global level), but also downwards towards cities, recognising the importance to act locally and in a coordinated fashion^(9)^. We deem that this is especially true when the most transformative albeit most challenging, leverage points are not episodic changes (i.e. those bound in time and resources), but rather those that require paradigm shifts in values and belief systems. Participants in this study emphasised the power of community cohesion and collective consumer choice to ignite social movements, in line with previous reports to protect the environment^(49)^. This could be attributed to the participation from grassroot efforts and citizen leaders in the GMB workshop, but we also acknowledge the well-documented deep mistrust in government among Puerto Ricans^(32)^. Attention needs to be paid to how transformative change is achieved and, as Baker *et al.* posit, ‘the who or what might enable or impede those changes going forward’^(9)^. Olson and Eoyang have declared that transformative systems change can emerge from the alignment between the stakeholders’ current values and beliefs with values and beliefs that will be needed to make changes in policies, procedures and other regulatory processes^(50)^. Foster-Fillman et al postulate that stakeholders’ current endogenous values (attitudes, beliefs and assumptions) along with regulatory procedures (policies and procedures), available resources (human and social capital) and dominant operations (power and control structures) maintain the status quo and impede the systems to transform^(19)^. The structures and processes that have been put in place by social, political and economic forces continue to reinforce a distribution of resources and power that maintains the status quo in the current food system(s)^(9)^. The extent to which the values of stakeholders align with the current system or with the envisioned system that protects nutrition security seems to be an important determining factor in starting paradigm shifts and remains to be evaluated.

### Strengths and limitations

The findings of this study must be interpreted within the stated scope of this work and the context of its methodological limitations and strengths. First, the model depicted is qualitative and therefore represents what the stakeholders considered relevant and important. The limits of the systems represented in the hybrid CLD also represent the perspectives of what was important to the stakeholders present in the workshop. Therefore, it is not meant to be an objective representation of the system(s) and their function but rather emphasises examining the subjective perspectives of those who participated, and, by definition, it is limited by who was present and whose perspectives were represented during the workshop. In this study, we sought to obtain participation from the multiple levels and niches within a system, including actors, organisations and system layers by drawing on the insider perspective of the community and practitioner partners of this study. However, it is possible that we drew boundaries too wide or even too narrow which may place limits on our understanding and ability to leverage change. Second, only one GMB session was conducted on this topic, meaning that there is potential to continue to expand on our findings by bringing together more stakeholders to get a better sense of the issues surrounding production, as well as strengthening systems thinking capacity. We did not gather follow-up information on the actions and networks that emerged among stakeholders after the workshop, which limits our ability to assess its overall impact, or to evaluate the partnerships and networks of participants prior or after the workshop.

### Conclusions

Transformative adaptations in our local food systems are needed to address the inter-related crises in human health, ecological degradation and local food production economy. The findings from this participatory CBSD contribute to understanding the complex interconnections between systems and to identify potential transformative adaptations in PR, emerging as intervention points developed through system-level understanding shared across synergistic communities of action.

## Supporting information

Colón-Ramos et al. supplementary material Colón-Ramos et al. supplementary material

## Supplementary material

For supplementary material accompanying this paper visit https://doi.org/10.1017/S1368980025101080

## References

[1] Mozaffarian D , Fleischhacker S & Andrés JR (2021) Prioritizing nutrition security in the US. JAMA 325, 1605–1606. 33792612 10.1001/jama.2021.1915

[2] Hammond RA & Dubé L (2012) A systems science perspective and transdisciplinary models for food and nutrition security. Proc Natl Acad Sci USA 109, 12356–12363. 22826247 10.1073/pnas.0913003109 PMC3411994

[3] Fanzo J , Bellows AL , Spiker ML et al. (2021) The importance of food systems and the environment for nutrition. Am J Clin Nutr 113, 7–16. 33236086 10.1093/ajcn/nqaa313 PMC7717136

[4] Forrester J & Warfield J (1973) World Dynamics, 2nd ed. Cambridge, MA: Warren-Allen Press, Inc.

[5] Swinburn BA , Kraak VI , Allender S et al. (2019) The global syndemic of obesity, undernutrition, and climate change: the Lancet Commission Report. Lancet 393, 791–846. 30700377 10.1016/S0140-6736(18)32822-8

[6] Garrity K , Krzyzanowski Guerra K , Hart H et al. (2014) Local food system approaches to address food and nutrition security among low-income populations: a systematic review. Adv Nutr 15, 100156. 10.1016/j.advnut.2023.100156 PMC11031423 38616069

[7] Baker P , Hawkes C , Wingrove K et al. (2018) What drives political commitment for nutrition? A review and framework synthesis to inform the United Nations Decade of Action on Nutrition. BMJ Glob Health 3, e000485. 10.1136/bmjgh-2017-000485 PMC5841521 29527338

[8] Alvaro C , Jackson LA , Kirk S et al. (2011) Moving Canadian governmental policies beyond a focus on individual lifestyle: some insights from complexity and critical theories. Health Promot Int 26, 91–99. 20709791 10.1093/heapro/daq052 PMC3033735

[9] Baker P , Lacy-Nichols J , Williams O et al. (2021) The political economy of healthy and sustainable food systems: an introduction to a special issue. Int J Health Policy Manag 10, 734–744. 34836454 10.34172/ijhpm.2021.156 PMC9309973

[10] Holman IP , Brown C , Carter TR et al. (2019) Improving the representation of adaptation in climate change impact models. Reg Environ Change 19, 711–721. 30956567 10.1007/s10113-018-1328-4 PMC6418063

[11] Blanco V , Brown C , Holzhauer S et al. (2017) The importance of socio-ecological system dynamics in understanding adaptation to global change in the forestry sector. J Environ Manage 196, 36–47. 28284136 10.1016/j.jenvman.2017.02.066

[12] Intergovernmental Panel on Climate Change (2022) Impacts, Adaptation and Vulnerability. Contribution of Working Group II to the Sixth Assessment Report of the Intergovernmental Panel on Climate Change. https://www.ipcc.ch/report/sixth-assessment-report-working-group-ii/ (accessed 04 June 2025).

[13] Brennan LK , Sabounchi NS , Kemner AL et al. (2015) Systems thinking in 49 communities related to healthy eating, active living, and childhood obesity. JPHMP 21, S55. 10.1097/PHH.0000000000000248 25828223

[14] Chebli P , Đoàn LN , Thompson RL et al. (2023) Identifying opportunities for collective action around community nutrition programming through participatory systems science. Cancer Causes Control 34, 1043–1058. 37481755 10.1007/s10552-023-01751-6 PMC10979368

[15] Wopereis TM , Dijkstra C , Wierda JJ et al. (2024) Systems thinking for local food environments: a participatory approach identifying leverage points and actions for healthy and sustainable transformations. Health Res Policy Syst 22, 101. 39135050 10.1186/s12961-024-01199-3 PMC11318250

[16] Mui Y , Ballard E , Lopatin E et al. (2019) A community-based system dynamics approach suggests solutions for improving healthy food access in a low-income urban environment. PLoS One 14, e0216985. 31086409 10.1371/journal.pone.0216985 PMC6516673

[17] Rouwette EAJA , Korzilius H , Vennix JAM et al. (2011) Modeling as persuasion: the impact of group model building on attitudes and behavior. Syst Dyn Rev 27, 1–21.

[18] Baker P , Brown AD , Wingrove K et al. (2019) Generating political commitment for ending malnutrition in all its forms: a system dynamics approach for strengthening nutrition actor networks. Obes Rev 20, 30–44. 31245905 10.1111/obr.12871

[19] Foster-Fishman PG , Nowell B & Yang H (2007) Putting the system back into systems change: a framework for understanding and changing organizational and community systems. Am J Community Psychol 39, 197–215. 17510791 10.1007/s10464-007-9109-0

[20] Hovmand PS (2014) Introduction to community-based system dynamics. In Community Based System Dynamics, pp. 1–16 [ PS Hovmand , editor]. New York, NY: Springer.

[21] Trías Monge J (1997) Puerto Rico: The Trials of the Oldest Colony in the World. New Haven, CT: Yale University Press.

[22] Carro-Figueroa V (2002) Agricultural decline and food import dependency in Puerto Rico: a historical perspective on the outcomes of postwar farm and food policies. Caribbean Stud 30, 77–107.

[23] Benach J , Díaz MR , Muñoz NJ et al. (2019) What the Puerto Rican hurricanes make visible: chronicle of a public health disaster foretold. Soc Sci Med 238, 112367. 31213368 10.1016/j.socscimed.2019.112367

[24] Dietz JL (1989) Historia económica de Puerto Rico (Economic history of Puerto Rico). San Juan: Ediciones Huracán.

[25] United States Department of Agriculture National Agricultural Statistics (2022) Census by State – Puerto Rico. https://www.nass.usda.gov/Publications/AgCensus/2022/Full_Report/Census_by_State/Puerto_Rico/index.php (accessed 04 June 2025).

[26] Perez R (2005) The State and Small-Scale Fisheries in Puerto Rico. Florida: University Press of Florida.

[27] Rivera Santiago CJ , López Rivera JR & Zayas Moró N (2022) *Economic Analysis Report 2020–2021*. Puerto Rico: PR Bureau of Labor Statistics. www.mercadolaboral.pr.gov (accessed 01 June 2025).

[28] Marrero A , Haneuse S , Golden CD et al. (2023) Neo-traditional and industrialized dietary patterns coexist and are differentially associated with cardiometabolic health among adults in Puerto Rico. J Nutr 153, 3259–3269. 37689268 10.1016/j.tjnut.2023.09.003 PMC10687615

[29] Rodriguez Ayuso IR (2017) *Encuesta sobre consumo de alimentos en establecimientos de comida rapida (Survey about consumption of foods in fast food establishments)*. Instituto de Estadisticas de Puerto Rico. www.estadisticas.pr (accessed 08 August 2024).

[30] Bezares N , McClain AC , Tamez M et al. (2023) Consumption of foods away from home is associated with lower diet quality among adults living in Puerto Rico. J Acad Nutr Diet 123, 95–108.e10. 35738537 10.1016/j.jand.2022.06.009 PMC9763551

[31] Colón-Ramos U , Rodríguez-Ayuso I , Gebrekristos HT et al. (2017) Transnational mortality comparisons between archipelago and mainland Puerto Ricans. J Immigrant Minority Health 19, 1009–1017. 10.1007/s10903-016-0448-5 27334006

[32] Cruz A (2020) Essay: Rethinking Government, A New Commonwealth-Muni Partnership. https://oversightboard.pr.gov (accessed 01 June 2025).

[33] Hunter JM & Arbona SI (1995) Paradise lost: an introduction to the geography of water pollution in Puerto Rico. Soc Sci Med 40, 1331–1355. 7638643 10.1016/0277-9536(94)00255-r

[34] Lugo AE (2019) Social-Ecological-Technological Effects of Hurricane María on Puerto Rico: Planning for Resilience under Extreme Events. Cham: Springer International Publishing.

[35] García-López GA (2018) The multiple layers of environmental injustice in contexts of (un)natural disasters: the case of Puerto Rico post-hurricane Maria. Environ Justice 11, 101–108.

[36] Febles N & Felix G (2020) Hurricane María, agroecology, and climate change resiliency. In *Climate Justice and Community Renewal: Resistance and Grassroots Solutions*, 1st ed. [ B Tokar , T Gilbertson , editors]. London: Routledge.

[37] Leiserowitz A , Verner M , Goddard E et al. (2023) International Public Opinion on Climate Change, 2023. Yale Program on Climate Change Communication. https://climatecommunication.yale.edu (accessed 04 June 2024).

[38] Santos-Burgoa C , Sandberg J , Suárez E et al. (2018) Differential and persistent risk of excess mortality from Hurricane Maria in Puerto Rico: a time-series analysis. Lancet Planet Health 2, e478–e488. 30318387 10.1016/S2542-5196(18)30209-2

[39] Dávila PJC (2022) Secretario de Agricultura: Puerto Rico producirá el 50 % de lo que consume para el 2024 (Secretary of Agriculture: Puerto Rico will produce 50% of what it consumes by 2024). El Nuevo Dia. June 9, 2022. www.elnuevodia.com (accessed 01 June 2025).

[40] Hovmand P , Rouwette EAJA , Andersen D et al. (2011) Scriptapedia: a handbook of scripts for developing structured group model building sessions. In *Proceedings of the 29th International Conference of the System Dynamics Society*, 24–28 Jul, pp. 1476–1491. Washington, DC: System Dynamics Society.

[41] Tolley EE , Ulin PR , Mack N et al. (2016) Qualitative Methods in Public Health: A Field Guide for Applied Research. Hoboken, NJ: John Wiley & Sons.

[42] Meadows D (2008) Thinking in Systems: A Primer. White River Junction, VT: Chelsea Green Publishing.

[43] Malhi L , Karanfil Ö , Merth T et al. (2009) Places to intervene to make complex food systems more healthy, green, fair, and affordable. J Hunger Environ Nutr 4, 466–476. 23173029 10.1080/19320240903346448 PMC3489112

[44] Meadows D (1999) Leverage Points: Places to Intervene in a System. The Sustainability Institute. donellameadows.org (accessed 04 May 2024).

[45] Guariguata L , Hickey GM , Murphy MM et al. (2023) Understanding the links between human health, ecosystem health, and food systems in Small Island Developing States using stakeholder-informed causal loop diagrams. PLOS Glob Public Health 3, e0001988. 37725624 10.1371/journal.pgph.0001988 PMC10508617

[46] Leng KH , Yaroch AL , Nugent NB et al. (2022) How does the Gus Schumacher Nutrition Incentive Program work? A theory of change. Nutrients 14, 2018. 35631159 10.3390/nu14102018 PMC9146513

[47] Johnston LM , Matteson CL & Finegood DT (2014) Systems science and obesity policy: a novel framework for analyzing and rethinking population-level planning. Am J Public Health 104, 1270–1278. 24832406 10.2105/AJPH.2014.301884 PMC4056198

[48] Béné C (2022) Why the great food transformation may not happen – a deep-dive into our food systems’ political economy, controversies and politics of evidence. World Dev 154, 105881.

[49] Kleres J & Wettergren Å (2017) Fear, hope, anger, and guilt in climate activism. Soc Mov Stud 16, 507–519.

[50] Olson EE & Eoyang GH (2001) Facilitating Organization Change: Lessons from Complexity Science. San Francisco, CA: Jossey- Bass Publishers.

