# Supplementary material for: Structures and systems that promote nutrition security and climate adaptation in Puerto Rico: results from community-based system dynamics
Source: Public Health Nutr. 2025 Sep 15;28(1):e162. doi: 10.1017/S1368980025101080 (PMC12516607; doi:10.1017/S1368980025101080)

**Appendix A**: **The three Causal Loop Diagrams created by participants of a Group Model Building Workshop to develop a shared understanding of system dynamics driving nutrition security in the face of climate change held in San Juan, PR, March 2023.** The CLDs were created in groups each with multiple stakeholder perspectives of the problem to offer new insights into improving food & nutrition security, while remaining grounded in participant experiences.


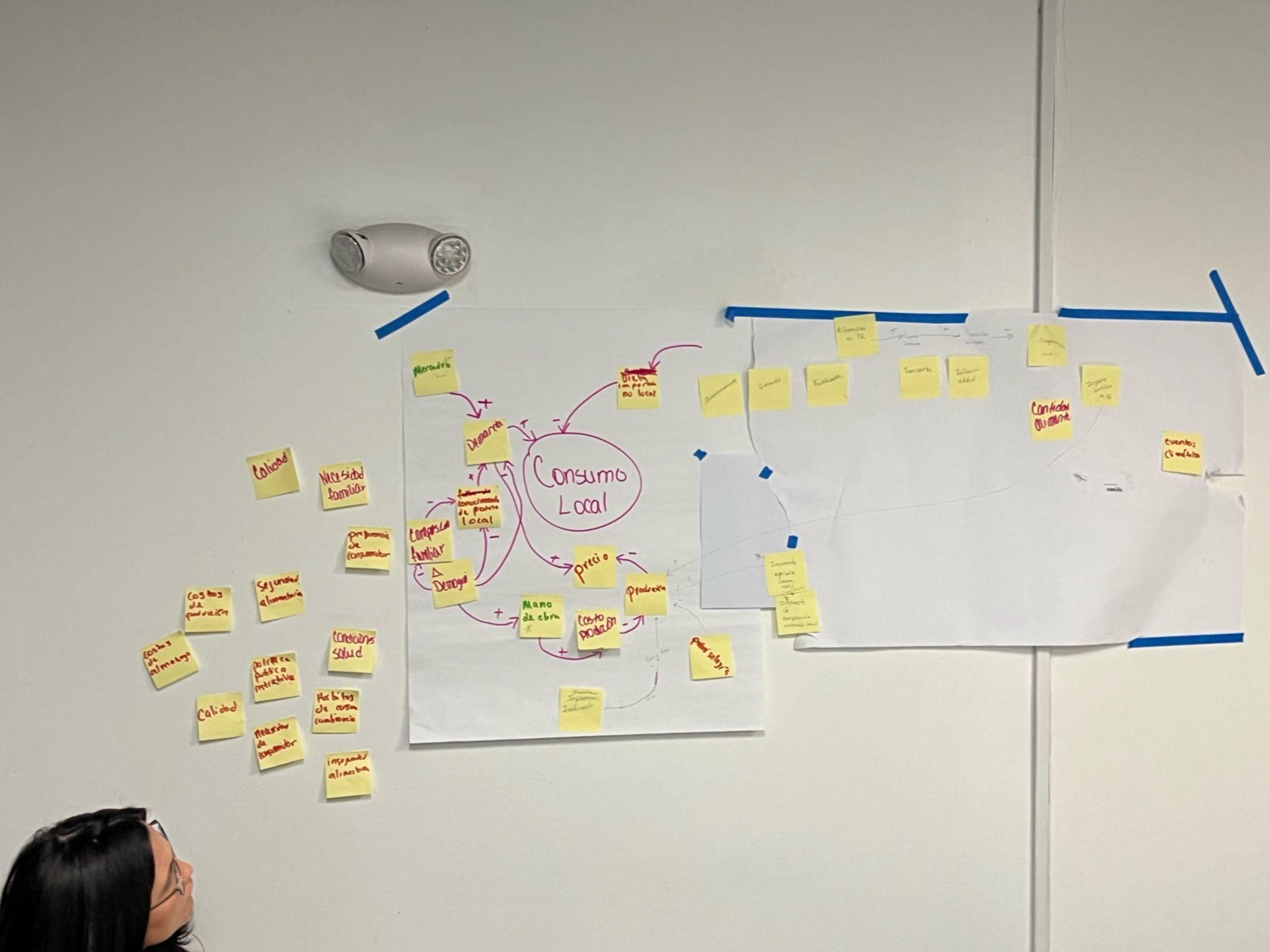


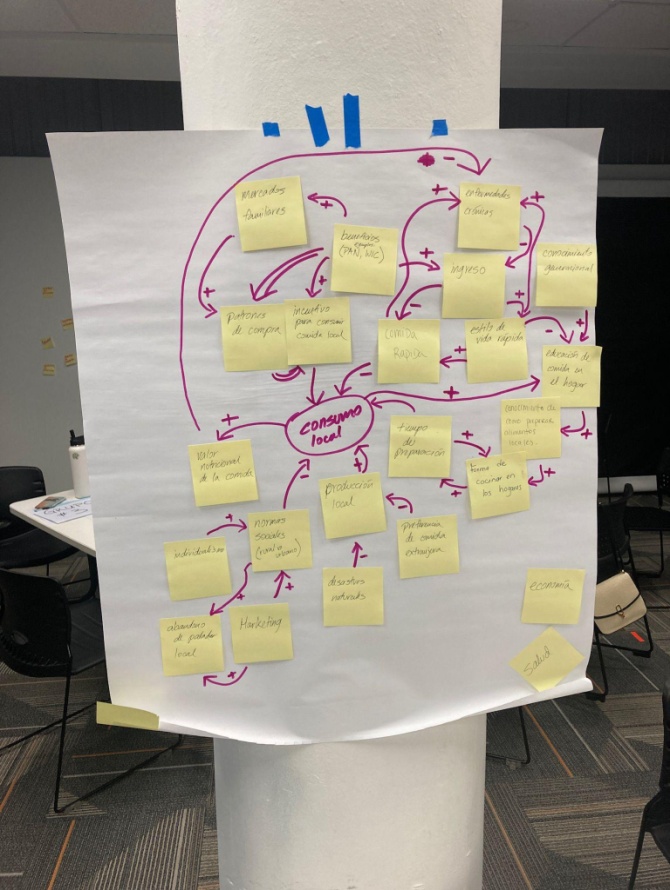

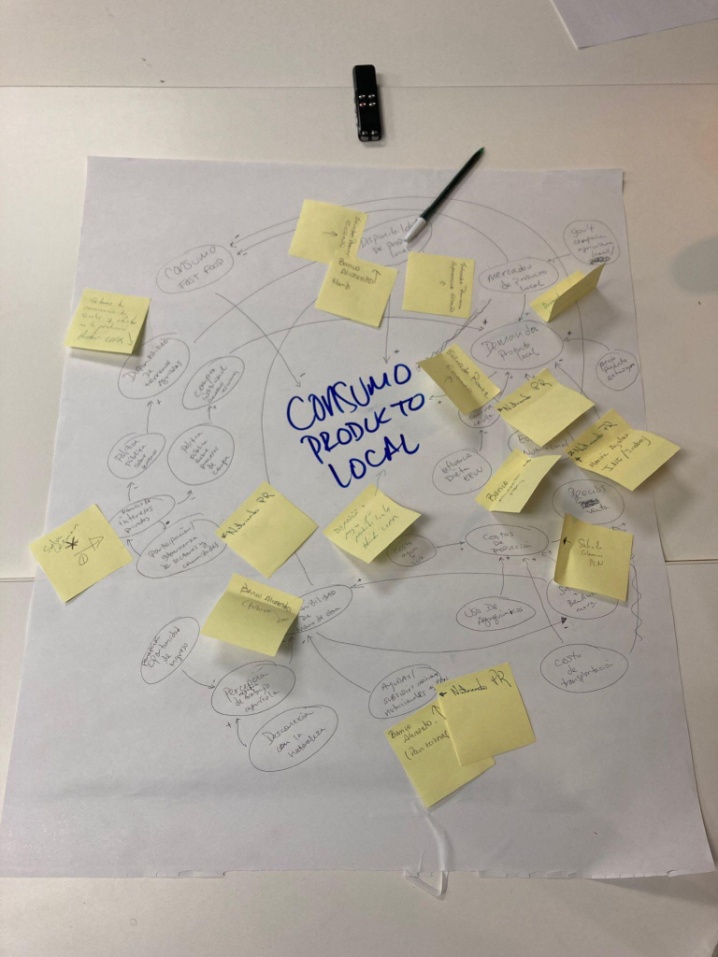

Supplement: Colón-Ramos et al. supplementary material [file S1368980025101080sup001.docx]
